# US Nitrous Oxide Mortality

**DOI:** 10.1001/jamanetworkopen.2025.22164

**Published:** 2025-07-30

**Authors:** R. Andrew Yockey, Rachel A. Hoopsick

**Affiliations:** 1 Department of Public Health, School of Applied Sciences, University of Mississippi, Oxford; 2 Department of Health and Kinesiology, College of Applied Health Sciences, University of Illinois at Urbana-Champaign, Champaign

## Abstract

This cohort study describes national trends in nitrous oxide poisoning mortality in the US from 2010 to 2023.

## Introduction

More than 13 million people in the US report using the inhalant drug nitrous oxide (also known as laughing gas or whippets) in their lifetime.^1^ Nitrous oxide misuse poses serious health risks, such as oxygen deprivation, which may result in hypoxia, neurological damage, and even death.^2^ Emergency department visits related to nitrous oxide misuse are increasing,^3^ but comprehensive data on the frequency of poisonings and associated mortality in the US remain sparse.^2,3^ This study examines national trends in nitrous oxide poisoning mortality in the US.

## Methods

This cohort study was deemed exempt from review and informed consent by the University of Mississippi institutional review board because this is not human participants research and the dataset does not contain any identifying information. This study is reported following the Strengthening the Reporting of Observational Studies in Epidemiology (STROBE) reporting guideline.

We obtained final death certificate data from 2010 to 2023 using the Centers for Disease Control and Prevention Wide-Ranging Online Data for Epidemiologic Research multiple causes of death database.^4^ We examined the annual number of deaths involving nitrous oxide and the age-adjusted overdose mortality rates per 100 000 population among US residents aged 15 to 74 years. We included causes of death in the following categories (*International Statistical Classification of Diseases and Related Health Problems, Tenth Revision* [*ICD-10*] codes): accidental/unintentional poisoning (X42, X43, X44, X47, X49), intentional self-poisoning/suicide (X62, X63, X64, X67, X69), assault/homicide (X85), and undetermined intent (Y12, Y13, Y14, Y17, Y19). Additionally, nitrous oxide deaths included deaths with a contributing cause of poisoning by nitrogen oxides (T59.0), but this code also includes deaths attributed to other binary compounds of oxygen and nitrogen.

We used Joinpoint regression to quantify crude mortality trends in nitrous oxide poisoning, applying permutation tests to detect significant changes in trends by fitting joined linear segments on a logarithmic scale to aggregated data. This method estimates the annual percentage change (APC) for each segment of the trend. We also estimated 95% CIs with each APC. Analyses were conducted with Joinpoint Regression Program version 5.4.0.0. The trend regression coefficient was considered a statistically significant increase or decrease if 2-sided *P* < .05. Analyses were conducted with Joinpoint Regression software version 5.4.0.0 (National Cancer Institute at the National Institutes of Health). Data were analyzed from March 1 to 18, 2025.

## Results

From 2010 to 2023, there was a total of 1240 deaths attributable to nitrous oxide poisoning among people aged 15 to 74 years in the US, with 23 deaths observed in 2010 and 156 deaths observed in 2023 (Table). Across the study period, the crude rate of deaths involving nitrous oxide poisoning ranged from a low of 0.0100 (95% CI, 0.0064 to 0.0151) deaths per 100 000 population in 2010 to a high of 0.0622 (95% CI, 0.0524 to 0.0719) deaths per 100 000 population in 2023 (Figure). There was a statistically significant change in the crude nitrous oxide poisoning rate from 2010 to 2018 (APC, 24.5% [95% CI, 18.6% to 51.2%]; *P* < .001). However, from 2019 to 2023, there was no crude mortality rate increase (APC, 3.6% [95% CI, −20.8% to 14.3%]; *P* = .67).

**Table.** Number and Rate of Deaths Involving Nitrous Oxide Poisoning Among People Aged 15 to 74 Years by Year, 2010 to 2023^a^

Year	Deaths, No.	Crude mortality rate, No. (95% CI) per 100 000 population
2010	23	0.0100 (0.0064 to 0.0151)
2011	28	0.0121 (0.0080 to 0.0175)
2012	29	0.0124 (0.0083 to 0.0178)
2013	37	0.0157 (0.0111 to 0.0217)
2014	38	0.0160 (0.0113 to 0.0219)
2015	47	0.0196 (0.0144 to 0.0260)
2016	120	0.0497 (0.0408 to 0.0586)
2017	90	0.0370 (0.0297 to 0.0454)
2018	127	0.0520 (0.0429 to 0.061)
2019	149	0.0608 (0.0510 to 0.0706)
2020	124	0.0504 (0.0415 to 0.0593)
2021	123	0.0494 (0.0406 to 0.0581)
2022	149	0.0596 (0.0501 to 0.0692)
2023	156	0.0622 (0.0524 to 0.0719)

^a^ Data extracted from the Centers for Disease Control and Prevention Wide-Ranging Online Data for Epidemiologic Research multiple causes of death database.^4^

**Figure.**
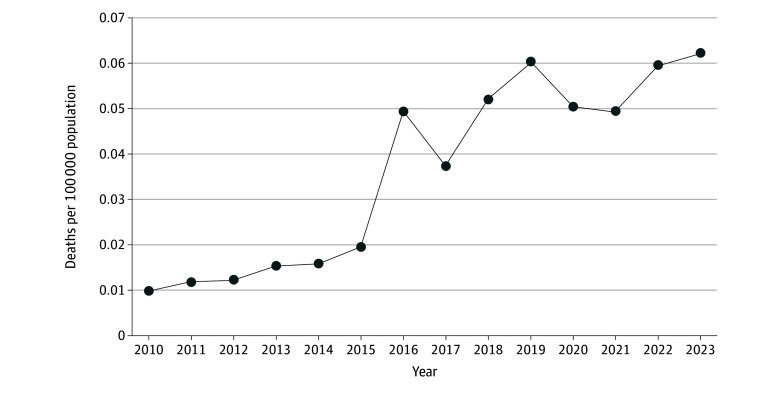
Crude Nitrous Oxide Poisoning Mortality Rate in the US From 2010 to 2023

## Discussion

Nitrous oxide–related mortality increased markedly from 2010 to 2023. This upward trend was significant through 2018 but plateaued from 2019 to 2023. These patterns align with increasing recreational use, particularly among adolescents and young adults.^5,6^ Contributing factors may include increased availability and low cost, although these were not assessed directly.

This study has some limitations, including suppression of small cell counts, exclusion of nonresident deaths, and potential underreporting if nitrous oxide was not tested. Lack of disaggregated data limits analyses by age or intent. Despite these constraints, findings underscore a growing public health concern and the need for enhanced surveillance and prevention efforts.

## Notes

Supplement.
Data Sharing Statement
