# Supplementary material for: US Nitrous Oxide Mortality
Source: JAMA Netw Open. 2025 Jul 30;8(7):e2522164. doi: 10.1001/jamanetworkopen.2025.22164 (PMC12311712; doi:10.1001/jamanetworkopen.2025.22164)
Supplement: Supplement. — Data Sharing Statement [file jamanetwopen-e2522164-s001.pdf]

Data Sharing Statement

Yockey. US Nitrous Oxide Mortality. *JAMA Netw Open*. Published August 01, 2025.
doi:10.1001/jamanetworkopen.2025.22164

Data
Data available: Yes
Data types: Data (not involving human participants)
How to access data: https://wonder.cdc.gov
When available: With publication

Supporting Documents
Document types: None

Additional Information
Who can access the data: anyone requesting the data
Types of analyses: Trends
Mechanisms of data availability: with investigator support
